# Lenalidomide-Induced Pure Red Cell Aplasia

**DOI:** 10.4274/Tjh.2013.0207

**Published:** 2014-03-05

**Authors:** Tuphan Kanti Dolai, Shyamali Dutta, Prakas Kumar Mandal, Sandeep Saha, Maitreyee Bhattacharyya

**Affiliations:** 1 NRS Medical College and Hospital, Department of Hematology, Kolkata, India

**Keywords:** Lenalidomide, PRCA, MSD

## TO THE EDITOR

Pure red cell aplasia (PRCA) is a bone marrow failure disorder. Several drugs have been reported as having induced PRCA, but lenalidomide-induced PRCA is rarely reported. Here we present a patient with myelodysplastic syndrome (MDS) who developed PRCA after treatment with lenalidomide.

A 47-year-old male presented with a history of weakness with effort intolerance for 5 months. Examination showed pallor and mild splenomegaly. Blood count showed Hb level of 86 g/L, total WBC count of 5.2x10^9^/L, normal differential counts, and platelet count of 223x10^9^/L. MCV was 101 fL and reticulocyte count was 1%. Serum chemistries were normal. Bone marrow aspiration and biopsy results showed mild hypercellularity with evidence of megaloblastic erythropoiesis and dyserythropoiesis. Cytogenetics showed loss of the Y chromosome. Iron stores were normal. Serum B12, folic acid, and serum ferritin levels were normal. Tests for antinuclear antibodies, HIV-1 and HIV-2 antibodies, hepatitis B surface antigen, and anti-hepatitis C virus were negative. Expressions of CD55 and CD59 on granulocytes were in the normal range. He received vitamin B12 with no success. With the diagnosis of MDS with refractory anemia, he was started on lenalidomide at 5 mg daily.

In the next 3 weeks, he presented with severe weakness and the Hb level dropped to 53 g/L with normal WBC and platelets. Reticulocyte count was <1%. Concentrated RBCs were transfused and he was re-evaluated. A repeat bone marrow aspiration and biopsy revealed severely depressed erythropoiesis (erythroid precursors of <5%) and dysplastic megakaryocytes. Parvovirus antibody (IgM) was negative. As there were no other inciting factors for PRCA, we stopped the lenalidomide. Oral prednisolone at 1 mg/kg was started and given for 8 weeks, with which Hb levels returned to the pre-PRCA range. Repeated aspiration showed normal erythropoiesis with dyserythropoiesis. Steroids were tapered over the next 2 months and the patient is still under follow-up and doing well, with Hb levels ranging between 80 and 95 g/L without transfusion support. Informed consent was obtained.

Here we report a patient with MDS who was treated with lenalidomide and developed PRCA, which improved with withdrawal of the drug and treatment with corticosteroids. In the literature to date, only one case has been reported of lenalidomide-induced PRCA [1]. Re-challenging the patient with lenalidomide might have given additional evidence about this phenomenon, but this was not justifiable.

The most common offensive drugs are erythropoietin, phenytoin, isoniazid, azathioprine, and zidovudine [2]. The pathogenesis of development of drug-induced PRCA is unknown; it may be due to direct effects of the drug per se, induction of autoimmunity, or specific inhibitory effects on DNA synthesis, probably at the step of deoxyribonucleotide formation [3]. Although there are no guidelines for the management of drug-induced PRCA, discontinuation and steroids may be of benefit [4]. 

## CONFLICT OF INTEREST STATEMENT

The authors of this paper have no conflicts of interest, including specific financial interests, relationships, and/or affiliations relevant to the subject matter or materials included. 
